# Triiron Complexes Featuring Azadiphosphine Related to the Active Site of [FeFe]-Hydrogenases: Their Redox Behavior and Protonation

**DOI:** 10.3390/molecules29143270

**Published:** 2024-07-10

**Authors:** Ahmad Hobballah, Catherine Elleouet, Philippe Schollhammer

**Affiliations:** Laboratoire de Chimie, Electrochimie Moléculaire et Chimie Analytique, UMR 6521 CNRS-Université de Bretagne Occidentale, CS 93837–6 Avenue Le Gorgeu, CEDEX 3, 29238 Brest, France; ahmad.hobballah@univ-brest.fr

**Keywords:** triiron complexes, metal–sulfur, carbonyl, thiolate, azadiphosphine, redox behaviors, protonation processes, hydrogenases, bioinspiration

## Abstract

The design of iron clusters featuring a bimetallic core and several protonation sites in the second coordination sphere of the metal centers is important for modeling the activity of polymetallic active sites such as the H-cluster of [FeFe]-hydrogenases. For this purpose, the syntheses of complexes [Fe_3_(CO)_5_(*κ*^2^-P^Ph^_2_N^R^_2_)(*μ*-pdt)_2_] (R = Ph (**1**), Bn (**2**)) and [Fe_3_(CO)_5_(*κ*^2^-P^Ph^_2_N^R^_2_)(*μ*-adt^Bn^)(*μ*-pdt)] (R = Ph (**3**), Bn (**4**)) were carried out by reacting hexacarbonyl precursors [Fe_2_(CO)_6_(*µ*-xdt)] (xdt = pdt (propanedithiolate), adt^Bn^ (azadithiolate) with mononuclear complexes [Fe(*κ*^2^-pdt)(CO)_2_(*κ*^2^-P^Ph^_2_N^R^_2_)] (P^Ph^_2_N^R^_2_ = (P^Ph^CH_2_N^R^CH_2_)_2_, R = Ph, Bn) in order to introduce amine functions, through well-known P^Ph^_2_N^R^_2_ diphosphine, into the vicinity of the triiron core. The investigation of the reactivity of these triiron species towards the proton (in the presence of CF_3_SO_3_H) and the influence of the pendant amines on the redox properties of these complexes were explored using spectroscopic and electrochemical methods. The protonation sites in such triiron clusters and their relationships were identified. The orientation of the first and second protonation processes depends on the arrangement of the second coordination sphere. The similarities and differences, due to the extended metal nuclearity, with their dinuclear counterparts [Fe_2_(CO)_4_(*κ*^2^-P^Ph^_2_N^R^_2_)(*μ*-pdt)], were highlighted.

## 1. Introduction

**Scheme 1:**
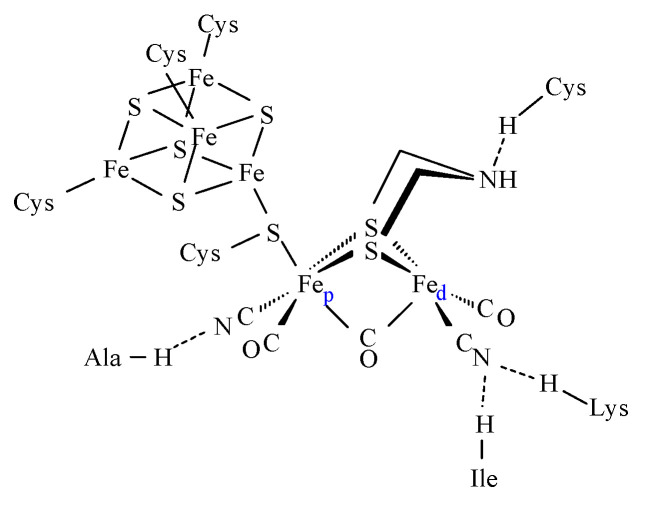
H-cluster, taken from the structure of CpI (PDB 3C8Y) [1].

According to the physiological context in the cell, [Fe-Fe] hydrogenases can either provide electrons by oxidizing molecular hydrogen or remove them through the production of H_2_, thus balancing the redox potential of the cell or providing it with energy [1,2,3]. Some shadows on the catalytic cycle of these enzymes remain so far [4,5,6,7,8], but it is strongly suggested that the transfer of protons is carried out by pendant bases, especially the amine function at the bridge-head of the diiron center of the active site (H-cluster) [9,10]. The H-cluster consists of a diiron center bridged by an azadithiolate group and linked to a cubane [Fe4S4] cluster via a bridging cysteine sulfur (Scheme 1) [1,3]. The secondary amine is proposed to serve as a proton relay between the distal iron atom (Fe_d_) and the amino acid residues of the catalytic proton transfer pathway. Moreover, its protonation is known to drive the transfer of one electron from the cuboidal [Fe4S4] cluster to the diiron center via a proton-coupled electron transfer (PCET) mechanism [4]. The main role of the [Fe4S4] cluster is to be an electron reservoir wherein proton transfers also occur to equilibrate the charges [11]. Recent works showed that the redox-state transitions in [Fe-Fe] hydrogenases are explained by selective PCET at the diiron site or at the [Fe4S4] cluster [12,13,14,15,16,17,18], with the directionality being influenced by hydrogen-bonding changes (pH-dependent). The design of iron clusters with a bimetallic core and several protonation sites in the second coordination sphere of the polymetallic core is important because it should offer the possibility (i) to generate multiple competitive protonation pathways, (ii) to mimic a proton channel around a diiron site, (iii) to favorably balance the charges to trigger electron transfers or PCET, and (iv) to check the protic versus redox non-innocence of the functions. In this context, the cyclic diphosphines (P^Ph^CH_2_N^R^CH_2_)_2_ (P^Ph^_2_N^R^_2_ with R = Ph or Bn) are interesting ligands for cooperative bifunctionality because, on the one hand, they afford the possibility of providing pendant amine functions in the second coordination sphere of complexes and, on the other hand, they are able to impose a geometric constraint for the positioning of the amine relative to the diiron center in the same way as the azadithiolate bridge in the H-cluster, but unlike monodentate amine-functionalized ligands. The use of such chelates in bioinspired mononuclear nickel or iron complexes allowed for the improvement of the efficiency of the species as catalysts for the activation of H_2_ or its production [19].

A series of diiron complexes with different combinations of dithiolato bridges (dithiolato = pdt, pdt^Et2^, pdt^Bn2^) and diphosphines P^Ph^_2_N^R^_2_ with R = Ph or Bn was recently described [20]. This study showed the slight effect of the bridge-head group of the dithiolate on the electronic properties of the compounds and demonstrated that the protonation of the amine and the NH^+^→Fe_2_(*μ*H)^+^ prototropic process is strongly dependent on the nature of the azadiphosphine. The proton transfer occurs with the NPh group while it is arrested at N-protonated species with the NBn group. Non-existent in the case of P^Ph^_2_N^Bn^_2_/CF_3_SO_3_^–^ combination, the prototropic transfer occurs after adding Na(BAr^F^_4_) salt in the medium. The influence of the ion pairing and consequently the nature of counterions on the thermodynamics and kinetics of proton transfer reactions of metallic hydrides is known [21,22,23,24,25,26,27]. The formation of the hydride species is favored with a counterion such as BAr^F^_4_^−^ or BPh_4_^−^ while the ammonium form is stabilized with BF_4_^−^ by the NH…F bonds [25,26,27]. Alongside the work reported on diiron complexes, a study of triiron complexes [Fe_3_(CO)_5_(*κ*^2^-dppe)(*μ*-xdt)(*μ*-pdt)] (xdt = pdt, pdt^Et2^, pdt^Bn2^, adt^Bn^; dppe = 1,2-bis(diphenylphosphino)ethane) was carried out [28]. Depending on the strength of the acid, bridging hydrido species are formed while the protonation of the amine function arises when pdt^R2^ bridges are replaced by an azadithiolate one.

A limited number of quasi-linear triiron species were reported in literature [28,29,30,31,32,33,34]. Yet, these models are of interest because they allow for the study of the effect that an increase of the metal nuclearity may have on the redox behavior and the protonation pathway of close dinuclear structures. In this work, triiron complexes bearing a combination of a propanedithiolate and azadithiolate bridge with cyclic azadiphosphines (P^Ph^CH_2_N^R^CH_2_)_2_ were synthesized and characterized. Then, their reactivity towards the proton and their redox behavior were studied.

## 2. Results and Discussion

### 2.1. Synthesis, Spectroscopic, and Structural Characterization of Complexes [Fe_3_(CO)_5_(κ^2^-P^Ph^_2_N^R^_2_)(μ-pdt)_2_] (R = Ph (***1***), Bn (***2***)), [Fe_3_(CO)_5_(κ^2^-P^Ph^_2_N^R^_2_)(μ-adt^Bn^)(μ-pdt)] (R = Ph (***3***), Bn (***4***))

**Scheme 2:**
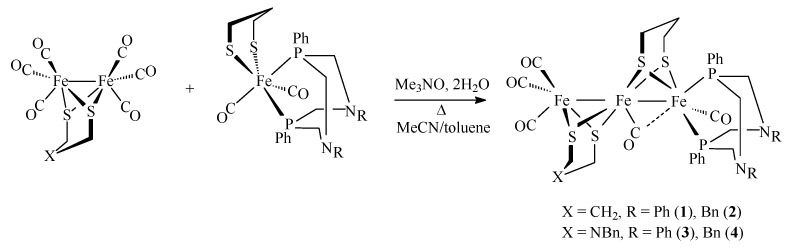
Synthesis of triiron clusters **1**–**4**.

The triiron complexes [Fe_3_(CO)_5_(*κ*^2^-P^Ph^_2_N^R^_2_)(*μ*-pdt)_2_] (R = Ph (**1**), Bn (**2**)) and ([Fe_3_(CO)_5_(*κ*^2^-P^Ph^_2_N^R^_2_)(*μ*-adt^Bn^)(*μ*-pdt)] (R = Ph (**3**), Bn (**4**)) were synthetized by the reaction of [Fe_2_(CO)_6_(*μ*-dithiolate)] (dithiolate = pdt or adt^Bn^) with [Fe(*κ*^2^-pdt)(CO)_2_(*κ*^2^-P^Ph^_2_N^R^_2_)] (see preparation and characterization in SI) in the presence of two equivalents of Me_3_NO.2H_2_O in a mixture of a toluene and acetonitrile solvent (4:1) for 2 h (Scheme 2) [34].

All the complexes were characterized using IR and NMR spectroscopies and elemental analysis (Appendix A). The IR spectra of the trinuclear complexes **1**–**4** present the expected patterns, which consist in three bands viz. two strong bands at ca 2020 cm^−1^, 1950 cm^−1^, and a weak absorption at ca 1830 cm^−1^ (Table 1 and Figure S2a). The observation of two doublets in the ^31^P{^1^H} NMR spectra (CDCl_3_) (Table 1 and Figure S2b) suggests a basal–apical position of the diphosphine [32], which is confirmed from the X-ray analysis of complexes **1** and **4**.

**Figure 1:**
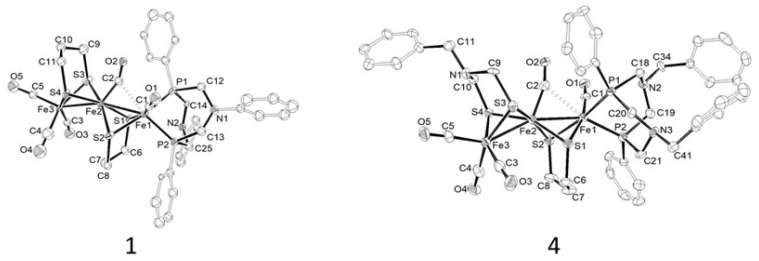
ORTEP views of **1**, **4**.

Single crystals of complex **4**, suitable for X-ray crystallography analysis (Table S1), were grown from the diffusion of hexane in a CH_2_Cl_2_ solution at −20 °C. The ORTEP view is displayed in Figure 1, while the selected crystallographic data are given in Table 2. Complex **4** adopts a typical structure for complexes [Fe_3_(CO)_5_(*κ*^2^-diphosphine)(*μ*-dithiolate)_2_] [28,29,30,31,32,33,34]. It is composed of a [3Fe4S] core, in which the iron centers are bridged by dithiolate bridges in anti-arrangement. The environment of each iron atom is, if the metal–metal interaction is not considered, a square-based pyramid. The two phosphorus atoms of the diphosphine P^Ph^_2_N^Bn^_2_ coordinate to one iron atom in a basal–apical position (Fe1-P1 = 2.206(2) Å, Fe1-P2 = 2.179(3) Å), which is different from the dibasal coordination mode of the 1,2-bis(diphenylphosphino)ethane (dppe) ligand observed in analogous complexes. The phosphorus, carbon, and the nitrogen atoms of the diphosphine form two contiguous cycles with the iron atom (FePCNCP), one of which adopts a boat conformation and the other which adopts a chair conformation. The bite angle P1-Fe1-P2 of 81.30(10)° is smaller than that observed for the dppe (~ 87°) in tri- and diiron complexes [34,35]. The triiron skeleton is slightly bent (Fe1-Fe2-Fe3 = 155.85(7)°) with iron–iron distances Fe1-Fe2 of 2.5459(19) Å and Fe2-Fe3 of 2.5693(19) Å, which is consistent with two single Fe-Fe bonds. A carbonyl group lies in a semi-bridging position (C2-Fe2 = 1.765(10) Å, C2-Fe1 = 2.403(9) Å, Fe2-C2-O2 = 160.8(8)°) (Figure 1, Table 2). The benzyl group carried by the nitrogen atom in the azadithiolate bridge is oriented equatorially with respect to the metallacycles {FeSCNCS}. A poor X-ray analysis of single crystals of complex **1**, obtained in similar crystallization conditions, confirmed the overall structure of complex **1** (Figure 1, Tables S2 and S3), and will not be further discussed.

### 2.2. Electrochemical Behavior of Complexes in the Absence of Protons

**Figure 2:**
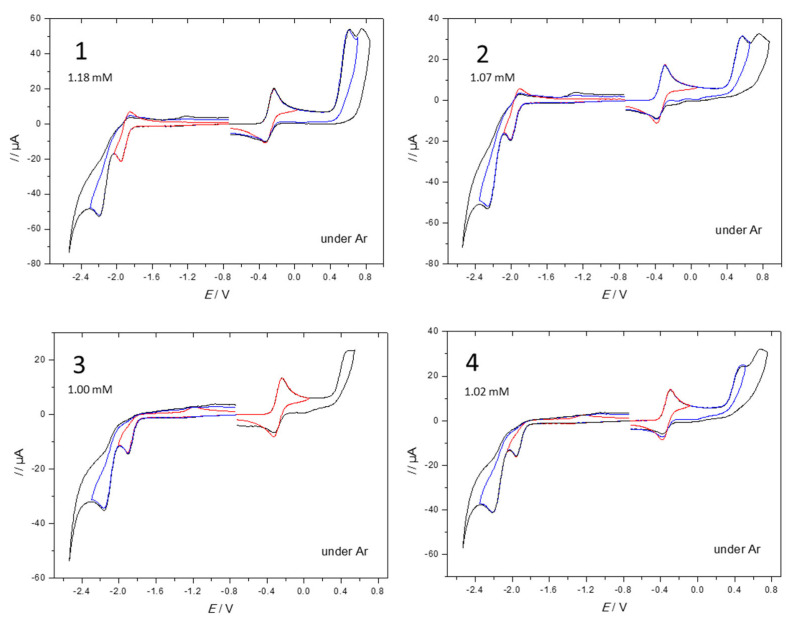
Cyclic voltammetry of the triiron related complexes **1**–**4** in CH_2_Cl_2_-[NBu_4_][PF_6_] 0.2 M at 0.2 V s^−1^.

The electrochemical properties of triiron complexes **1**–**4** were studied in CH_2_Cl_2_-[NBu_4_][PF_6_] 0.2 M with cyclic voltammetry (CV), the working electrode being a vitreous carbon (Figure 2). Potentials, given versus the couple (Fc^+^/Fc), are gathered in Table 3.

The single-electron oxidation of the complexes possessing the same P^Ph^_2_N^R^_2_ ligand takes place at the same potential. The same trend is observed for their single-electron reduction. Whatever the dithiolate bridge, the difference between the oxidation peak potentials (Δ*E*_p_^ox1^) of the P^Ph^_2_N^Ph^_2_ and P^Ph^_2_N^Bn^_2_ analogues is ca 60 mV, like the difference between the reduction peak potentials (Δ*E*_p_^red1^). This observation suggests that these shifts are rather due to the nature of the substituents in the diphosphine P^Ph^_2_N^R^_2_ than that of the dithiolate bridges. These observations also suggest that the amine does not coordinate to one iron center during the oxidation and the reduction. Indeed, the behavior does not correspond to that observed in the case of the diiron complex [Fe_2_(CO)_4_(*κ*^2^-dmpe)(*µ*-adt^Bn^)], for which it was demonstrated that, during the oxidation, an agostic/anagostic {C-H …Fe} interaction takes place after the one-electron transfer and an interaction between the amine function and the iron atoms after the two-electron transfer [36,37].

The anions of the bridged azadithiolate triiron complexes **3**,**4** are less stable than those of their bridged propanedithiolate analogues **1**,**2**. Indeed, at 0.2 V s^−1^, these latter exhibit a reversible reduction, whereas the reduction of the triiron compounds possessing an adt^Bn^ bridge is irreversible. In the case of [Fe_3_(CO)_5_(*κ*^2^-dppe)(*µ*-adt^Bn^)(*µ*-pdt)] [28], the reduction is also irreversible at low scan rates. At 0.2 V s^−1^, signs of reversibility are observed but not improved at higher scan rates, suggesting an ECC mechanism. If such a mechanism is involved during the reduction of **3**,**4**, this implies that the rate constants of the chemical reactions are much faster, since the reduction remains irreversible when the scan rate increases. The nature of the bridge carried by the iron atoms not coordinated to the ligand P^Ph^_2_N^R^_2_ impacts the potential of the second reduction observed in CV. Indeed, the difference (*E*_1/2_^red1^–*E*_p_^red2^) is 300 mV for **1**,**2** (complexes with a pdt bridge) and 260 mV for **3**,**4** (complexes with an adt^Bn^ bridge). This difference is 215 mV in the case of the complex [Fe_3_(CO)_5_(*κ*^2^-dppe)(*µ*-adt^Bn^)(*µ*-pdt)] [28], while no second reduction is observed on the cyclic voltammetry of the complex [Fe_3_(CO)_5_(*κ*^2^-dppe)(*µ*-pdt)_2_] [28]. The difference between the two oxidation peaks is ca 900 mV for **1**,**2** while it is ca 800 mV for their dppe analogues and complexes **3**,**4**.

For all triiron complexes **1**–**4**, the CV in oxidation under CO is similar to that observed under an inert atmosphere (Figure S3), which suggests that the chemical reaction involved in the EC or ECC mechanism under Ar is not a reversible loss of CO. The reduction under CO takes place according to an ECE mechanism, corresponding to the formation of an adduct between the reduced species and CO, which reduces to a potential that is less negative than that of the starting material. This behavior is reminiscent of that observed for the triiron complexes featuring a dppe ligand [28]. It is interesting to remember that the explanation for the slowness of the chemical reaction in the case of the complex [Fe_3_(CO)_5_(*κ*^2^-dppe)(*µ*-pdt)_2_] lies in the PPh_2_ de-coordination, allowing the thermodynamically favorable binding of CO. The rate is faster in the case of the [Fe_3_(CO)_5_(*κ*^2^-dppe)(*µ*-adt^Bn^)(*µ*-pdt)] complex. In the case of P^Ph^_2_N^R^_2_ complexes, the chemical reactions are even faster.

### 2.3. Behavior of Complexes in the Presence of Protons

**Figure 3:**
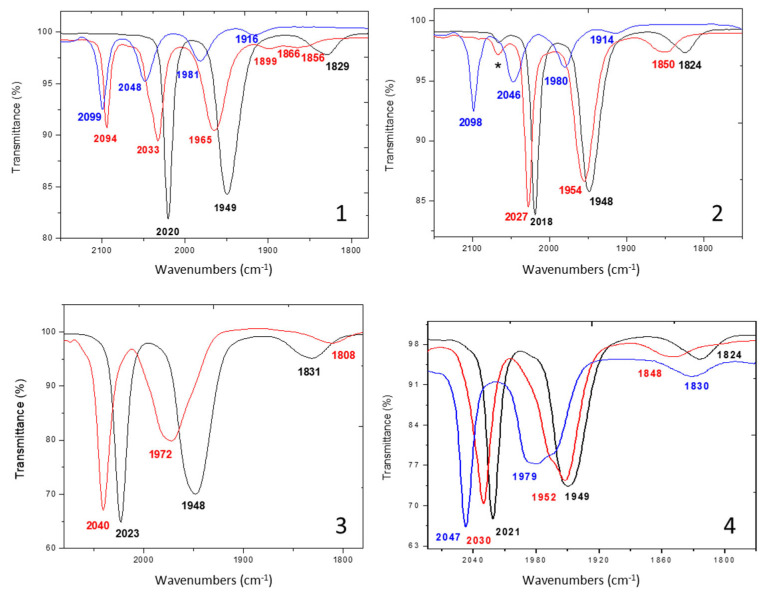
IR (CH_2_Cl_2_) spectra of complexes **1**–**4** at 25 °C in the presence of CF_3_SO_3_H (black curve = neutral complex, red curve = monoprotonated species, blue curve = doubly protonated species, * = impurity).

**Figure 4:**
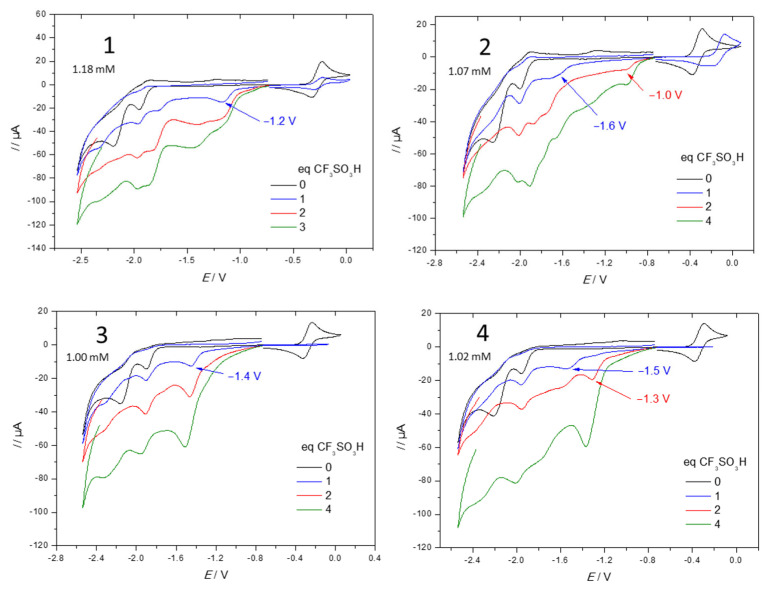
Cyclic voltammetry of complexes **1**–**4** in the presence of CF_3_SO_3_H in CH_2_Cl_2_-[NBu_4_][PF_6_] 0.2 M at v = 0.2 V s^−1^.

The protonation of complexes **1**–**4** with CF_3_SO_3_H was monitored in CH_2_Cl_2_ using IR spectroscopy (Figure 3) and the cyclic voltammograms of **1**–**4** in the presence of increasing amounts of this acid were recorded (Figure 4).

**Scheme 3:**
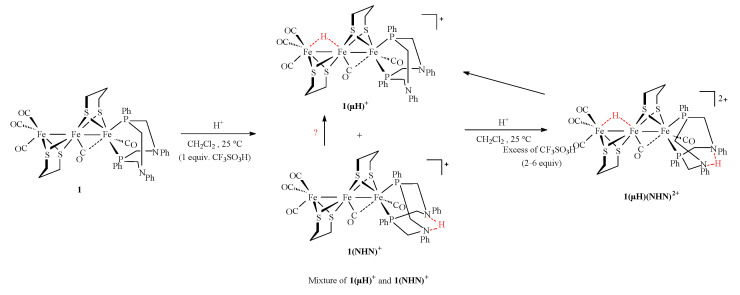
Protonation of the triiron cluster **1**.

The IR spectrum of complex **1** in the presence of one equivalent of CF_3_SO_3_H (Figure 3) shows three strong bands of terminal CO at 2094, 2033, and 1965 cm^−1^. Additional weak bands relative to the *µ*-CO are detected at 1899, 1866, and 1856 cm^−1^. The shift of ca 70 cm^−1^ of the higher ῡ(CO) band (2094 cm^−1^) with respect to those of the neutral complex (2020, 1949, 1829 cm^−1^) suggests the formation a bridging hydrido species, as it is observed for the protonation of [Fe_3_(CO)_5_(*κ*^2^-dppe)(*µ*-pdt)_2_] [28]. The unusual relative intensities of the ῡ(CO) bands for a bridging hydrido species indicates that, in fact, this pattern is due to the overlapping of bands of two compounds resulting from the formation of a bridging hydrido and a N-protonated species, **1(*µ*H)^+^** and **1(NHN)^+^**, respectively (Scheme 3). The observation of a third band in the µ-CO region suggests the existence of a third isomer, which is not further discussed. Based on the literature [28], the hydride is positioned between the two iron atoms not carrying the chelating ligand P^Ph^_2_N^Ph^_2_. The conversion of **1(NHN)^+^** into **1(*µ*H)^+^**, as it was proposed for the dinuclear complex [Fe_2_(CO)_4_(*κ*^2^-P^Ph^_2_N^Ph^_2_)(*µ*-pdt)] [20], could not be unambiguously observed. In the presence of an excess of acid, a further shift towards higher wavenumbers is observed, affording a well-defined ῡ(CO) band pattern (2099 (vs), 2048 (s), 1981 (m), and 1916(w) cm^−1^) (Figure 3), which indicates the formation of a doubly protonated species **1(*µ*H)(NHN)^2+^** (Scheme 3). Interestingly, the spontaneous deprotonation of **1(*µ*H)(NHN)^2+^** arose in the IR cell during the recording of the spectrum, which allowed the observation of the ῡ(CO) bands of a species, tentatively assigned to the bridged hydrido species **1(*µ*H)^+^** alone (2094 (vs), 2037 (s), 1971 (m) and 1900 (w)) (Figure S4), which is similar to that of **1(*µ*H)(NHN)^2+^**, but with a red shift of 5–10 cm^−1^. This shows the easy deprotonation of the doubly protonated species on the amine function. The decrease in the intensity of ῡ(CO) bands suggests the decomposition of the protonated species, which is observed after several hours of the stirring of the reaction mixture by the disappearance of the carbonyl bands. Unlike the other complexes **2**–**4**, in experimental CV recording conditions, the addition of more than one equivalent of trifluoromethanesulfonic acid (CF_3_SO_3_H) to [Fe_3_(CO)_5_(*κ*^2^-P^Ph^_2_N^Ph^_2_)(*µ*-pdt)_2_] (**1**) is required to allow its complete protonation (Figure 4), which would indicate that complex **1** is therefore less basic than **2**–**4**, unless the result is due to the kinetics. Moreover, the reduction potential of the protonated species is not the same (*E*_p_ = −1.2 V) as its adt^Bn^ analogue **3** (*E*_p_ = −1.4 V), suggesting that the site of protonation is different. The reduction of the doubly protonated species **1(*µ*H)(NHN)^2+^** is not observed in CV. Attempts to isolate protonated species have failed. Protonated species of triiron species **1**–**4** could not be isolated because of the lability of the protons, whatever the protonation site [28]. In order to obtain further information on the nature of these species and to support, when possible, hypotheses on their structures, in situ low temperature ^1^H and ^31^P{^1^H} NMR protonation experiments were performed. A solution of complex **1** was prepared in CD_2_Cl_2_ in the presence of three equivalents of CF_3_SO_3_H added at −80 °C. The ^1^H and ^31^P{^1^H} NMR spectra were recorded at various temperatures, starting at −80 °C (Figures S5 and S6). In the ^31^P{^1^H} NMR spectrum at −30 °C, only one set of signals, consisting in a doublet of a doublet, at 20.1 and 43.4 ppm, with a coupling constant of 74 Hz, was detected, which suggests a basal apical disposition of the diphosphine and recalls the range of chemical shifts observed for doubly protonated diiron species [Fe_2_(CO)_4_(*κ*^2^-P^Ph^_2_N^R^_2_H)(*μ*-pdt)(*µ*-H)](CF_3_SO_3_)_2_ [20]. The observation in the ^1^H NMR spectrum, at −30 °C, of singlets at 13.17 and −10.47 ppm, which are assigned to an ammonium proton and {Fe_2_(µH)} hydrido, respectively, suggests the formation of the doubly protonated species **1(*µ*H)(NHN)^2+^**, as it was proposed previously on the basis of IR monitoring experiments. Similarly to the protonation of the analogous compound [Fe_3_(CO)_5_(*κ*^2^-dppe)(*µ*-pdt)_2_], the value (ca −10 ppm) and the absence of the multiplicity of the hydrido signal, due to the phosphorus atoms of the diphosphine, indicate that the protonation arises at the Fe_2_ site, which does not bear the diphosphine.

**Scheme 4:**
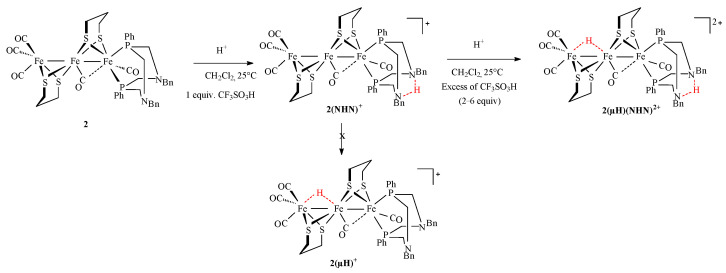
Protonation of the triiron cluster **2**.

The replacement of a phenyl with a benzyl group in the diphosphine P^Ph^_2_N^R^_2_ orients the protonation of complex **2** exclusively on the amine function, as it is reported for the dinuclear complex [Fe_2_(CO)_4_(*κ*^2^-P^Ph^_2_N^Bn^_2_)(*µ*-pdt)] [20]. Upon the addition of one equivalent of CF_3_SO_3_H to a solution of complex **2** in CH_2_Cl_2_, a slight shift at higher wavenumbers of the three carbonyl bands from 2018 (s), 1948 (s), and 1824 (w) cm^−1^ (Figure 3), to 2027(s), 1954(s), and 1850(w) cm^−1^ is observed, which is consistent with a N-protonation of complex **2** (Scheme 4). Further additions of CF_3_SO_3_H lead to a second protonation at the diiron center, affording the doubly protonated species **2(*µ*H)(NHN)^2+^**. A typical pattern with four bands is observed at 2098 (vs), 2046 (s), 1980 (m), and 1914 (w) cm^−1^. The reduction peak of the N-protonated species **2(NHN)^+^** occurs at −1.6 V. The formation of a doubly protonated species **2(*µ*H)(NHN)^2+^** is detected on the CV of complex **2** after the addition of two equivalents of acid by the presence of a new peak at −1.0 V, i.e., a shift of 0.9 V with respect to the unprotonated complex. Such a shift can be attributed to the fact that a dicationic species, simultaneously carrying a proton and a hydride, is formed [38]. Otherwise, the potential −1.0 V is reminiscent with the potential −1.1 V obtained for the reduction of the doubly protonated species, (*µ*H)(NH)^2+^, of the diiron complex [Fe_2_(CO)_4_(*κ*^2^-P^Ph^_2_N^Bn^_2_)(*µ*-pdt)]; the difference between the two values probably reflects the difference of nuclearity. ^1^H and ^31^P{^1^H} NMR protonation experiments were performed in the same way as for compound **1**. The ^1^H and ^31^P{^1^H} NMR spectra of compound **2** were recorded in CD_2_Cl_2_ in the presence of three equivalents of CF_3_SO_3_H at various temperatures, starting at −80 °C (Figures S7 and S8). The ^31^P{^1^H} NMR spectrum at −30 °C shows, in addition to a set of signals, consisting in a doublet of a doublet, at 22.4 and 42.3 ppm with a coupling constant of 89 Hz, which is assigned to **2(*µ*H)(NHN)^2+^**, an additional doublet of a doublet at 54.7 and 33.0 ppm with a coupling constant of 50.6 Hz. Based on IR monitoring, this species is assigned to the N-monoprotonated form **2(NHN)^+^**. The observation in the ^1^H NMR spectrum at −30 °C of singlets at 11.20 and −10.47 ppm also supports the formation of a doubly protonated species **2(*µ*H)(NHN)^2+^**. The signal of the proton at the nitrogen atom in **2(NHN)^+^** is tentatively assigned to a resonance ca 9 ppm.

**Scheme 5:**
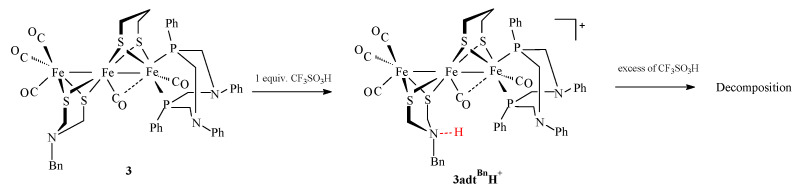
Protonation of the triiron cluster **3**.

In the case of the complex **3**, in which one pdt bridge is replaced by an adt^Bn^ group, the protonation occurs preferentially on the nitrogen atom of the azadithiolate bridge rather than the P^Ph^_2_N^Bn^_2_ diphosphine (Scheme 5). The IR spectrum of complex **3** in the presence of one equivalent of CF_3_SO_3_H shows three bands at 2040 (vs), 1972 (s), and 1808 (w) cm^−1^, which corresponds to an increase of about 15–20 cm^−1^ of the two higher ῡ(CO) bands (terminal carbonyl) compared to those of the neutral species **3** (2023 (vs), 1948 (s), 1831 (w) cm^−1^) (Figure 3). Conversely, the weak band at 1831 cm^−1^, which is assigned to the bridging carbonyl, is shifted at lower wavenumbers (ca 17 cm^−1^). This excludes the protonation at the P^Ph^_2_N^Ph^_2_ diphosphine. This red shift was observed for the complex [Fe_3_(CO)_5_(*κ*^2^-dppe)(*µ*-adt^Bn^)(*µ*-pdt)] in previous works [28]. It is proposed to be due to a hydrogen interaction between the *µ*-CO and the NH^+^ of the protonated azadithiolate bridge. Thus, it offers an interesting probe to identify the protonation site among the different amine functions. More, this behavior is reminiscent of that of the aminodithiolate ligand in the enzyme, which is believed to act as an inner-sphere hydrogen-bonding donor to a number of apical ligands at the distal iron [39,40]. Upon further additions of CF_3_SO_3_H, the degradation of the protonated species is observed.

**Scheme 6:**
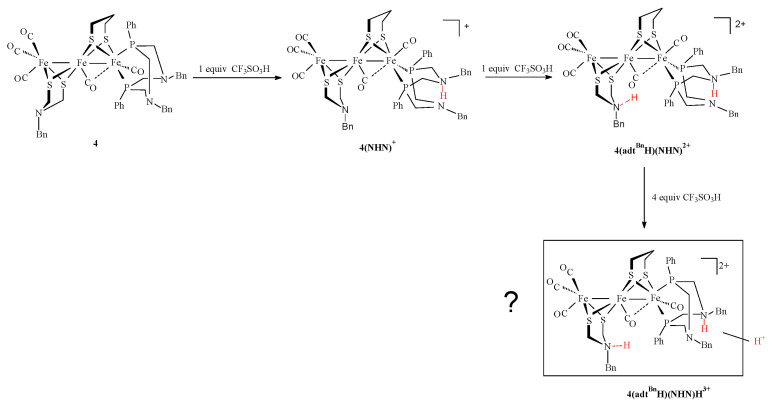
Protonation of the triiron cluster **4**.

The IR spectrum of the complex **4**, in which the diphosphine P^Ph^_2_N^Ph^_2_ has been replaced by P^Ph^_2_N^Bn^_2_ compared to complex **3**, after the addition of one equivalent of CF_3_SO_3_H, exhibits three bands at 2030 (vs), 1952 (s), and 1848 (w) cm^−1^, instead of 2021 (vs), 1949 (s), and 1824 (w) cm^−1^, which is in agreement with a protonation on the azadiphosphine (Figure 3). This **4(NHN)^+^** species reduces at −1.5 V. The addition of a supplementary equivalent of acid leads to the appearance of a new reduction peak located at a potential that is less negative (−1.3 V) than the previous one (−1.5 V), suggesting that a doubly protonated species is present in the solution (Scheme 6). This is confirmed by the IR spectrum, which shows that the three CO bands at 2030, 1952, and 1848 cm^−1^, are replaced by three new CO bands at 2047 (vs), 1979 (s), and 1830 (w) cm^−1^. The shift of the band of the bridging carbonyl at lower wavenumbers with respect to the monoprotonated species (1830 versus 1848 cm^−1^) indicates that the nitrogen atom of the azadithiolate bridge has been protonated, thus affording a doubly N-protonated species **4(adt^Bn^H)(NHN)^2+^**. Over time, similarly to **3adt^Bn^H^+^**, which evolves into **3adt^Bn^** (Figure S9), **4(adt^Bn^H)(NHN)^2+^** evolves slowly **4(NHN)^+^** in the IR cell. This shows the reversibility of the protonation step at the amine function of the azadithiolate bridge. Such reversible protonation is generally observed with di- or tri-iron N-protonated species with an azadithiolate bridge, unless there is a stabilization of the ammonium form. In the presence of a further excess of CF_3_SO_3_H, an additional evolution of the IR spectrum is observed. The new CO bands at 2056 (vs), 1997 (s), 1969 (sh), and 1861 (w) cm^−1^ are due to a third protonation in the second sphere of coordination or a tautomerization (proton migration) (Figure S10). The overall decrease in the intensity of the ῡ(CO) bands may result in part from the decomposition of protonated species.

## 3. Materials and Methods

All the syntheses of complexes **1**–**4** were carried out under an inert atmosphere, using Schlenk techniques. Solvents were deoxygenated and dried according to standard procedures. 1,3,5,7-tetraphenyl-1,5-diaza-3,7-diphosphacyclooctane [41], 1,5-dibenzyl-3,7-diphenyl-1,5,-diaza-3,7-diphosphacycloctane [41], complexes [Fe(*κ*^2^-pdt)(CO)_2_(*κ*^2^-P^Ph^_2_N^R^_2_)] (R = Ph, Bn) [42] and [Fe_2_(CO)_6_(*µ*-dithiolate)] [43] were prepared according to reported/adapted procedures (see SI for complexes [Fe(*κ*^2^-pdt)(CO)_2_(*κ*^2^-P^Ph^_2_N^R^_2_)]. ^31^P{^1^H} NMR and ^1^H NMR spectra were recorded at room temperature or low temperature in CDCl_3_ on a Bruker AMX 400 spectrometer of the “Service général des plateformes, Université de Bretagne Occidentale, Brest*”* and referenced to SiMe_4_. The infrared spectra were recorded on a Perkin–Elmer spectrometer. Chemical analyses were performed by the “*Service de Microanalyse I.C.S.N.*”, Gif sur Yvette (France).

Electrochemical measurements were conducted using a PG-STAT 128 N Autolab or a µ-Autolab (type III) electrochemical analyzer, driven by GPES software. All the electrochemical studies were carried out in a conventional three-electrode cell under an inert atmosphere (argon) or CO atmosphere. The preparation and the purification of the supporting electrolyte [NBu_4_][PF_6_] were conducted as described previously [44]. The working electrode was a vitreous carbon disk of 0.3 cm in diameter, polished with alumina prior to use. A platinum wire was used as a counter electrode. The reference electrode was the Ag|Ag^+^ electrode; however, all the potentials (text, tables, and figures) are quoted against the (Fc^+^/Fc) couple; ferrocene was added as an internal standard at the end of the experiments.

Crystal data for **1**, **4**, and **5** were collected on an Oxford Diffraction X-Calibur-2 CCD diffractometer, equipped with a jet cooler device and graphite-monochromated Mo-Kα radiation (λ = 0.71073 Å). The structure was solved and refined using standard procedures [45,46]. The deposition number CCDC 2352159, 2352160 contains the supplementary crystallographic data for **1** and **4**, respectively. These data can also be obtained free of charge from the Cambridge Crystallographic Data Centre via www.ccdc.cam.ac.uk/data_request/cif, accessed on 30 April 2024.

## 4. Conclusions

**Scheme 7:**
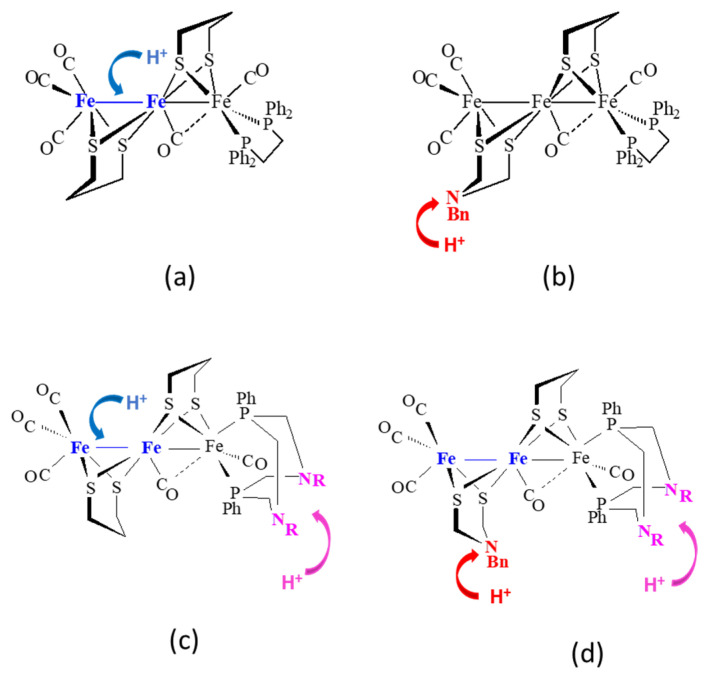
Different protonation sites (**a**–**d**) in triiron species depending on the nature of the dithiolate bridges and the diphosphines.

We previously showed that in the case of the triiron complex [Fe_3_(CO)_5_(*κ*^2^-dppe)(*μ*-pdt)_2_], only one site is protonated at the FeFe bond, which does not bear the diphosphine (Scheme 7) [28]. The replacement of the *µ*-pdt group in this diiron site with a *µ*-adt^Bn^, as it is found in [Fe_3_(CO)_5_(*κ*^2^-dppe)(*μ*-adt^Bn^)(*μ*-pdt)], orients the protonation to the nitrogen atom of this bridge, and no further protonation has been evidenced in both cases (Scheme 7) [28]. In this work, an additional protonation site is introduced into the vicinity of the Fe3S4 core through the diphosphine P^Ph^_2_N^R^_2_, the strength of which is modulated by the choice of the R group (R = Ph or Bn). NBn amine functions are protonated first when they are present in the complexes. In complex **2**, the P^Ph^_2_N^Bn^_2_ is selectively protonated with respect to the FeFe site and in complex **3**, the adt^Bn^ group is selectively protonated with respect to the P^Ph^_2_N^Ph^_2_ diphosphine. When the adt^Bn^ bridge and the P^Ph^_2_N^Bn^_2_ diphosphine are present together, as it is in complex **4**, the first protonation arises at the diphosphine site, because of a favorable stabilization of the N-protonated form through a N-H…N interaction. Concurrent protonations are observed in complex **1** at the diphosphine P^Ph^_2_N^Ph^_2_ and the FeFe sites, affording N- and FeFe-protonated species. That is different from the protonation of the diiron compound [Fe_2_(CO)_4_(*κ*^2^-P^Ph^_2_N^Ph^_2_)(*µ*-pdt)] which leads, under the same reaction conditions, to the formation of a bridging hydride species, through a NHN^+^ -> *µ*H^+^ tautomerization [20]. This shows an effect of the intercalation of a {Fe(CO)(pdt)} moiety fragment between the {Fe(CO)_3_} and {FeCO(P^Ph^_2_N^R^_2_)} ends, which changes the relative disposition of the base sites. This reduces the basicity of the diiron site and limits or prevents the transfer of the proton from the P^Ph^_2_N^Ph^_2_ diphosphine to the protonable diiron core in complex **1** compared to [Fe^2^(CO)_4_(*κ*^2^-P^Ph^_2_N^Ph^_2_)(*µ*-pdt)]. Indeed, the diphosphine is no longer carried by the protonable FeFe site in the triiron species, unlike the FeFe site in the diiron counterpart**.** The addition of an excess of acid to [Fe_3_(CO)_5_(*κ*^2^-P^Ph^_2_N^R^_2_)(*µ*-pdt)_2_] (**1**–**2**) affords N-protonated, bridged hydrido triiron dication, very similarly to their diiron counterpart [Fe_2_(CO)_4_(*κ*^2^-P^Ph^_2_N^R^_2_)(*µ*-pdt)]. In the case of complex **3**, no doubly protonated species is formed in the presence of an excess of acid, which suggests that the diphosphine P^Ph^_2_N^Ph^_2_ in this monocationic species is not sufficiently basic to be protonated. Differently, a doubly NBn protonated species is detected with complex **4**. No tautomerization process from one proton site to another one has been evidenced. To resume, if the diiron site supports an azadithiolate bridge, the protonation of this latter inhibits a second protonation at the bimetallic center. The protonation of the diphosphine P^Ph^_2_N^R^_2_ does not prevent a second protonation at the diiron core or the azadithiolate bridge. Depending on the basicity of the amine in the azadiphosphine, the protonation at the metal center can be competitive with that of the diphosphine or arise only in the second protonation step. The protonation at the diiron site does not prevent the protonation of the diphosphine.

Finally, these triiron clusters can be viewed as an active bimetallic Fe2S2 core {Fe_2_(CO)_4_(*µ*-dithiolate)} at which a metallo ligand {Fe(*κ*^2^pdt)(CO)_2_(*κ*^2^-P^Ph^_2_N^R^_2_)} is linked. Several pendant bases can be positioned more or less close to the dinuclear active site, which orients and modulates the protonation processes. The design of more compact diiron systems which are in close proximity to several amine functions is now under investigation.

## Figures and Tables

**Table 1:** IR (CH_2_Cl_2_) and ^31^P{^1^H} NMR (CDCl_3_) data of complexes **1**–**4** at 25 °C.

Complex	IR (CH_2_Cl_2_, cm^−1^), ῡ(CO)	^31^P{^1^H} NMR (CDCl_3_, δ: ppm, *J*: Hz)
**1**	2020 (s), 1949 (s), 1829 (w)	56.6 (d, ^2^*J*_PP_ = 80.0), 40.6 (d, ^2^*J*_PP_ = 80.0)
**2**	2018 (s), 1948 (s), 1824 (w)	53.3 (d, ^2^*J*_PP_ = 81.0), 40.8 (d, ^2^*J*_PP_ = 81.0)
**3**	2023 (s), 1948 (s), 1831 (w)	57.0 (d, ^2^*J*_PP_ = 80.0), 40.6 (d, ^2^*J*_PP_ = 80.0)
**4**	2021 (s), 1949 (s), 1824 (w)	53.7 (d, ^2^*J*_PP_ = 81.5), 40.8 (d, ^2^*J*_PP_ = 81.5)

**Table 2:** Selected distances and bond angles for **4**.

4			
Distance (Å)		Angles (°)	
Fe1-Fe2	2.5459(19)	Fe1-Fe2-Fe3	155.85(7)
Fe2-Fe3	2.5693(19)	Fe2-C2-O2	160.8(8)
Fe1-CO_terminal_	1.767(11)	Fe1-C2-O2	125.7(7)
Fe3-CO_terminal_	1.732(12)–1.797(10)	Fe2-C2-Fe1	73.5(3)
Fe1-CO_bridging_	2.403(9)	Fe1-Fe2-C2	64.8(3)
Fe2-CO_bridging_	1.765(10)	P1-Fe1-P2	81.30(10)
Fe1-P1	2.206(2)		
Fe1-P2	2.179(3)		

**Table 3:** Oxidation and reduction potentials of complexes **1**–**4** in CH_2_Cl_2_−[NBu_4_][PF_6_] 0.2 M at 0.2 V s^−1^.

Complex	Oxidation	Reduction
**1**	*E*_1/2_^ox1^ = −0.28 V; *E*_p_^ox2^ = 0.61 V	*E*_1/2_^red1^ = −1.90 V; *E*_p_^red2^ = −2.20 V
**2**	*E*_1/2_^ox1^ = −0.34 V; *E*_p_^ox2^ = 0.57 V	*E*_1/2_^red1^ = −1.95 V; *E*_p_^red2^ = −2.25 V
**3**	*E*_1/2_^ox1^ = −0.28 V; *E*_p_^ox2^ = 0.48 V	*E*_p_^red1^ = −1.90 V; *E*_p_^red2^ = −2.16 V
**4**	*E*_1/2_^ox1^ = −0.34 V; *E*_p_^ox2^ = 0.47 V	*E*_p_^red1^ = −1.96 V; *E*_p_^red2^ = −2.22 V

Potentials measured versus the couple (Fc^+^/Fc).

## Data Availability

The data presented in this study are available in Supplementary Materials.

## Supplementary Materials

The following supporting information can be downloaded at: https://www.mdpi.com/article/10.3390/molecules29143270/s1, Scheme S1. Synthesis of complexes [Fe(*κ*^2^-pdt)(CO)_2_(*κ*^2^-P^Ph^_2_N^R^_2_)] (R = Ph, Bn); Figure S1. IR (CH_2_Cl_2_) (a) and ^31^P^{1^H} NMR (CDCl_3_) (b) spectra of [Fe(*κ*^2^-pdt)(CO)_2_(*κ*^2^-P^Ph^_2_N^Ph^_2_)] at 25 °C; Figure S2. IR (CH_2_Cl_2_) (a) and ^31^P{^1^H} NMR (CDCl_3_) (b) spectrum of **1** at 25 °C; Figure S3. Comparison of CV of **1**–**4** under Ar and CO in CH_2_Cl_2_-[NBu_4_][PF_6_] 0.2 M at v = 0.2 V s^−1^; Table S1. Crystallographic data of [Fe_3_(CO)_5_(*κ*^2^-P^Ph^_2_N^Bn^_2_)(*μ*-adt^Bn^)(*μ*-pdt)] (**4**); Table S2. Crystallographic data of [Fe_3_(CO)_5_(*κ*^2^-P^Ph^_2_N^Ph^_2_)(*μ*-pdt)_2_] (**1**); Table S3. Selected distances and bond angles for **1**; Figure S4. IR spectra of **1** in CH_2_Cl_2_ in the presence of (a) 6 equiv of CF_3_SO_3_H and (b) after evolution in the IR cell; Figure S5. ^31^P{^1^H} NMR spectrum of **1** at 243 K in the presence of 3 equiv of CF_3_SO_3_H; Figure S6. ^1^H NMR spectrum of **1** at 243 K in the presence of 3 equiv of CF_3_SO_3_H; Figure S7. ^31^P{^1^H} NMR spectrum of **2** at 243 K in the presence of 3 equiv of CF_3_SO_3_H; Figure S8. ^1^H NMR spectrum of **2** at 243 K in the presence of 3 equiv of CF_3_SO_3_H; Figure S9. Monitoring in the IR cell of the evolution of **3adt^Bn^H^+^** in CH_2_Cl_2_ after 5 min (red curve) and 10 min (purple curve); IR spectrum of **3** in CH_2_Cl_2_ (black curve); Figure S10. IR (CH_2_Cl_2_) spectra of complex **4** in the presence of CF_3_SO_3_H: 0 equiv (blue curve), 2 equiv (blue curve) and 6 equiv (purple curve).

## Appendix A

*Preparation of complexes [Fe_3_(CO)_5_(κ^2^-P^Ph^_2_N^R^_2_)(μ-pdt)_2_] (R = Ph (***1***), Bn (***2***)), [Fe_3_(CO)_5_(κ^2^-P^Ph^_2_N^R^_2_)(μ-adt^Bn^)(μ-pdt)] (R = Ph (***3***), Bn (***4***))*

In a typical procedure, 0.2 g of [Fe_2_(CO)_6_(*µ*-dithiolate)] (0.518 mmol (pdt), 0.419 mmol (adt^Bn^) was dissolved in dry acetonitrile (50 mL). A total of 0.115 g (1.034 mmol) and 0.93 g (0.838 mmol) (2 equiv) of Me_3_NO·2H_2_O were added to the solutions of these complexes, respectively, and the resulting mixtures were stirred for 5 min. Then, one equivalent of [Fe(*κ*^2^-pdt)(CO)_2_(*κ*^2^-P^Ph^_2_N^R^_2_)], solubilized in toluene (100 mL) (0.348 g, 0.518 mmol, R = Ph), (0.293 g, 0.419 mmol, R= Bn), was added. The resulting solution was heated for 2 h at 90 °C. The solvent was then evaporated under a vacuum and the crude product was purified using column chromatography (silica gel) with mixtures of hexane–CH_2_Cl_2_ as the eluent. A first orange fraction, which corresponds to the unreacted diiron complexes, was eluted. Then, a second red fraction, identified as the side-product [{Fe_2_(CO)_5_(*μ*-pdt)}_2_(*μ*-P^Ph^_2_N^R^_2_)] [20], was collected in a small yield (5%). The main dark red band was then collected, and after the evaporation of the solvent, the triiron complexes **1**–**4** were obtained as red dark powders. Single crystals of complexes **1** and **4** were obtained from hexane–dichloromethane (3:1) solutions at −20 °C.

*Data for [Fe_3_(CO)_5_(κ^2^-P^Ph^_2_N^Ph^_2_)(μ-pdt)_2_] (* **1** *)*
  m = 0.26 g, yield = 52%
  IR (CH_2_Cl_2_, cm^−1^): ῡ(CO) = 2020 (s), 1949 (s), 1829 (w).
  ^31^P{^1^H} NMR (CDCl_3_, ppm): δ = 56.6 (d, ^2^*J*_PP_ = 80 Hz), 40. 6 (d, ^2^*J*_PP_ = 80 Hz).
  ^1^H NMR (CDCl_3_, ppm): δ = 7.89–6.95 (m, 20H, Ph), 4.55–3.79 (m, 8H, CH_2_, P^Ph^_2_N^Ph^_2_), 2.15–1.09 (m, 12H, CH_2_, pdt).
  Anal. Calcd (%) for C_39_H_40_Fe_3_N_2_O_5_P_2_S_4_
  (%) Theoretical: C = 48.07; H = 4.14; N = 2.87
  (%) Experimental: C = 48.13; H = 4.09; N = 2.74

*Data for [Fe_3_(CO)_5_(κ^2^-P^Ph^_2_N^Bn^_2_)(μ-pdt)_2_] (* **2** *)*
  m = 0.27 g, yield = 53%
  IR (CH_2_Cl_2_, cm^−1^): ῡ(CO) = 2018 (s), 1948 (s), 1824 (w).
  ^31^P{^1^H} NMR (CDCl_3_, ppm): δ = 53.3 (d, ^2^*J*_PP_ = 81 Hz), 40.8 (d, ^2^*J*_PP_ = 81 Hz).
  ^1^H NMR (CDCl_3_, ppm): δ = 7.65–7.19 (m, 20H, Ph), 4.09 (q(AB), ^2^*J*_HH_ = 13 Hz, 2H, NCH_2_Ph), 3.84 (q(AB), ^2^*J*_HH_ = 13 Hz, 2H, NCH_2_Ph), 3.77–2.88 (m, 8H, PCH_2_N), 2.37–1.07 (m, 12H, CH_2_, pdt).
  Anal. Calcd (%) for C_41_H_44_Fe_3_N_2_O_5_P_2_S_4_
  (%) Theoretical: C = 49.12; H = 4.42; N = 2.79
  (%) Experimental: C = 48.75; H = 4.41; N = 2.50

*Data for [Fe_3_(CO)_5_(κ^2^-P^Ph^_2_N^Ph^_2_)(μ-adt^Bn^)(μ-pdt)] (* **3** *)*
  m = 0.24 g, yield = 55%.
  IR (CH_2_Cl_2_, cm^−1^): ῡ(CO) = 2023 (s), 1948 (s), 1831 (w).
  ^31^P{^1^H} NMR (CDCl_3_, ppm): δ = 57.0 (d, ^2^*J*_PP_ = 80 Hz), 40.6 (d, ^2^*J*_PP_ = 80 Hz).
  ^1^H NMR (CDCl_3_, ppm): δ = 7.89–6.95 (m, 25H, Ph), 4.54–3.77 (m, 8H, P^Ph^_2_N^Ph^_2_), 3.45 (d(AB), ^2^*J*_HH_ = 14Hz, 2H, NCH_2_Ph), 3.15 (d, ^2^*J*_HH_ = 11Hz, 1H, SCH_2_N), 3.00 (d, ^2^*J*_HH_ = 11Hz, 1H, SCH_2_N), 2.51 (d, ^2^*J*_HH_ = 11Hz, 1H, SCH_2_N), 1.86 (d, ^2^*J*_HH_ = 11Hz, 1H, SCH_2_N), 2.12–1.28 (m, CH_2_, 6H, pdt).
  Anal. Calcd (%) for **3**, 2 CH_2_Cl_2_: C_47_H_49_Cl_4_Fe_3_N_3_O_5_P_2_S_4_
  (%) Theoretical: C = 45.69; H = 4.00; N = 3.40
  (%) Experimental: C = 46.17; H = 4.07; N = 3.39

*Data for [Fe_3_(CO)_5_(κ^2^-P^Ph^_2_N^Bn^_2_)(μ-adt)(μ-pdt)] (* **4** *)*
  m = 0.24 g, yield = 53%
  IR (CH_2_Cl_2_, cm^−1^): ῡ(CO) = 2021 (s), 1949 (s), 1824 (w).
  ^31^P{^1^H} NMR (CDCl_3_, ppm): δ = 53.7 (d, ^2^*J*_PP_ = 81.5 Hz), 40.8 (d, ^2^*J*_PP_ = 81.5 Hz).
  ^1^H NMR (CDCl_3_, ppm): δ = 7.64–7.13 (m, 25H, Ph), 4.04 (q(AB), ^2^*J*_HH_ = 13 Hz, 2H, NCH_2_Ph), 3.83 (q(AB), ^2^*J*_HH_ = 13 Hz, 1H, NCH_2_Ph), 3.75–3.27 (m, 8H, PCH_2_N), 3.42 (s, 2H, NCH_2_Ph), 3.12 (d, ^2^*J*_HH_ = 11.5 Hz, 1H, SCH_2_N), 2.91 (d, ^2^*J*_HH_ = 11.5 Hz, 1H, SCH_2_N), 2.50 (d, ^2^*J*_HH_ = 11.5 Hz, 1H, SCH_2_N), 2.32–1.26 (m, 6H, pdt), 1.78 (d, ^2^*J*_HH_ = 11.5 Hz, 1H, SCH_2_N).
  Anal. Calcd (%) for C_47_H_49_Fe_3_N_3_O_5_P_2_S_4_
  (%) Theoretical: C = 51.62; H = 4.52; N = 3.84.
  (%) Experimental: C = 50.83; H = 4.47; N = 3.68.

### Protonation Experiments

In a typical experiment, 10.5 mg (0.0107 mmol) of complex **1** was solubilized in 10 mL of degassed CH_2_Cl_2_. Successive additions of one equivalent of CF_3_SO_3_H (0.91 µL) were then made to reach a stoichiometry from one to six equivalents of acid relative to the initial quantity of the complex. The IR recording was performed after each additional acid addition. Similar experimental procedures were followed with complex **2** (9.2 mg, 0.00918 mol), complex **3** (10.5 mg, 0.0098 mmol), and complex **4** (10.8 mg, 0.00988 mmol).
