# Supplementary material for: Triiron Complexes Featuring Azadiphosphine Related to the Active Site of [FeFe]-Hydrogenases: Their Redox Behavior and Protonation
Source: Molecules. 2024 Jul 10;29(14):3270. doi: 10.3390/molecules29143270 (PMC11279172; doi:10.3390/molecules29143270)
Supplement: Supplementary file 1 [file molecules-29-03270-s001.zip › molecules-3078465-supplementary.pdf]

Supporting Information

Preparation and characterization of mononuclear complexes [Fe(κ^2-pdt)(CO)$_2$(κ^2-P$^{Ph}{}_2$N$^R{}_2$)] (R = Ph, Bn)

Scheme S1. Synthesis of complexes [Fe(κ^2-pdt)(CO)$_2$(κ^2-P$^{Ph}{}_2$N$^R{}_2$)] (R = Ph, Bn).

Figure S1. IR (CH_2Cl_2) (a) and ^{31}P{^{1}H} NMR ($CDCl_3$) (b) spectrum of [Fe(κ^2-pdt)(CO)$_2$(κ^2-P$^{Ph}{}_2$N$^{Ph}{}_2$)] at 25°C.

Figure S2. IR (CH_2Cl_2) (a) and ^{31}P{^{1}H} NMR ($CDCl_3$) (b) spectra of **1** at 25°C.

Figure S3. Comparison of CV of **1**-**4** under Ar and CO in CH_2Cl_2-[NBu$_4$][PF$_6$] 0.2 M at v = 0.2 V s^{-1}.

Table S1. Crystallographic data of [Fe$_3$(CO)$_5$(κ^2-P$^{Ph}{}_2$N$^{Bn}{}_2$)(μ-adt^{Bn})(μ-pdt)] (**4**)

Table S2. Crystallographic data of [Fe$_3$(CO)$_5$(κ^2-P$^{Ph}{}_2$N$^{Ph}{}_2$)(μ-pdt)$_2$] (**1**)

Table S3. Selected distances and bond angles for **1**.

Figure S4. IR spectra of **1** in CH_2Cl_2 in the presence of (a) 6 equiv of CF_3SO_3H and (b) after evolution in the IR cell.

Figure S5. ^{31}P{^{1}H} NMR spectrum of **1** at 243 K in the presence of 3 equiv of CF_3SO_3H

Figure S6. ^{1}H NMR spectrum of **1** at 243 K in the presence of 3 equiv of CF_3SO_3H

Figure S7. ^{31}P{^{1}H} NMR spectrum of **2** at 243 K in the presence of 3 equiv of CF_3SO_3H

Figure S8. ^{1}H NMR spectrum of **2** at 243 K in the presence of 3 equiv of CF_3SO_3H

Figure S9. Monitoring in the IR cell of the evolution of **3adt^{Bn}H$^+$** in CH_2Cl_2 after 5 min (red curve) and 10 min (purple curve) ; IR spectrum of **3** in CH_2Cl_2 (black curve).

Figure S10. IR (CH_2Cl_2) spectra of complex **4** in the presence of CF_3SO_3H : 0 equiv (blue curve), 2 equiv (blue curve) and 6 equiv (purple curve)

Preparation and characterization of mononuclear complexes [Fe(κ^2-pdt)(CO)$_2$(κ^2-P$^{Ph}{}_2$N$^R{}_2$)] (R = Ph, Bn)

The complexes [Fe(κ^2-pdt)(CO)$_2$(κ^2-P$^{Ph}{}_2$N$^R{}_2$)] **(**R = Ph, Bn) were prepared following adaptations of reported procedures [42] (Scheme S1). One equivalent of P$^{Ph}{}_2$N$^R{}_2$ (R =Ph (0.644 g, 1.42 mmol), Bn (0.684 g, 1.42 mmol)) was added to a solution of [FeI$_2$(CO)$_4$] (0.60 g, 1.42 mmol) in CH_2Cl_2. After 2h under vacuum, an IR monitoring showed the complete transformation into the complex [FeI$_2$(CO)$_2$(κ^2-P$^{Ph}{}_2$N$^R{}_2$)]. A solution of propanedithiol (0.18 mL, 1.79 mmol) and triethylamine (0.48 mL, 3.58 mmol), in 10 mL of CH_2Cl_2, were then added to the reaction solution. After 4h of stirring, the solvent was evaporated and the residue was purified by a column chromatography (silica gel) using CH_2Cl_2:THF (95:5) as eluent. A red band was collected and evaporated to dryness under vacuum which gives red powders of complexes [Fe(κ^2-pdt)(CO)$_2$(κ^2-P$^{Ph}{}_2$N$^R{}_2$)].

Data for [Fe(κ^2-pdt)(CO)$_2$(κ^2-P$^{Ph}{}_2$N$^{Ph}{}_2$)]:
m = 0.83 g, yield = 68%
IR (CH_2Cl_2, cm^{-1}) : ῡ(CO) = 2014 (s), 1966 (s).
^{31}P{^{1}H} NMR (CDCl$_3$, ppm) : δ = 39.8 (d, $^2J_{PP}$ = 129.5 Hz), 33.2 (d, $^2J_{PP}$ = 129.5 Hz).
^{1}H NMR (CDCl$_3$, ppm) : δ = 7.78 – 7.01 (m, 20H, Ph), [5.27 (m,1H), 4.71 (m,1H), 4.08 (m,3H), 3.93(m, 1H), 3.80 (m, 1H), 3.43 (m,1H)] (4 x CH$_2$, P$^{Ph}{}_2$N$^{Ph}{}_2$), 2.53, 2.47, 2.08 and 1.97 (m, 6H, CH$_2$, pdt).

Data for [Fe(κ^2-pdt)(CO)$_2$(κ^2-P$^{Ph}{}_2$N$^{Bn}{}_2$)] :
m = 0.80 g, yield = 67 %
IR (CH_2Cl_2, cm^{-1}) : ῡ(CO) = 2012 (s), 1963 (s).
^{31}P{^{1}H} NMR (CDCl$_3$, ppm) : δ = 38.5 (d, $^2J_{PP}$ = 130 Hz), 31.7 (d, $^2J_{PP}$ = 130 Hz).
^{1}H NMR (CDCl$_3$, ppm) : δ = 7.50 – 7.05 (m, 20H, Ph), 4.44 (m, 1H, PCH$_2$N), 4.04 (s, 2H, NCH$_2$Ph), 3.92 (m, 1H, PCH$_2$N), 3.69 (m, 2H, NCH$_2$Ph), 3.28-3.04 (m, 4H, PCH$_2$N) 3.91(m, 1H, PCH$_2$N), 2.60 (m, 1H, PCH$_2$N) (m, 8H, PCH$_2$N), 2.48, 2.41, 1.95 and 1.88 (m, 6H, pdt).

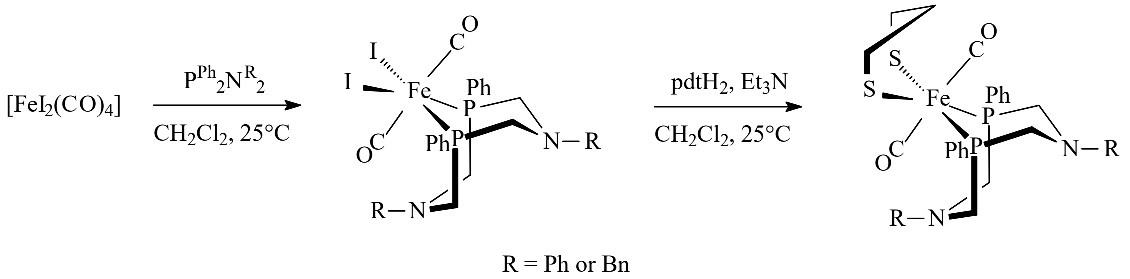

Scheme S1. Synthesis of complexes [Fe(κ^2-pdt)(CO)$_2$(κ^2-P$^{Ph}{}_2$N$^R{}_2$)] (R = Ph, Bn).

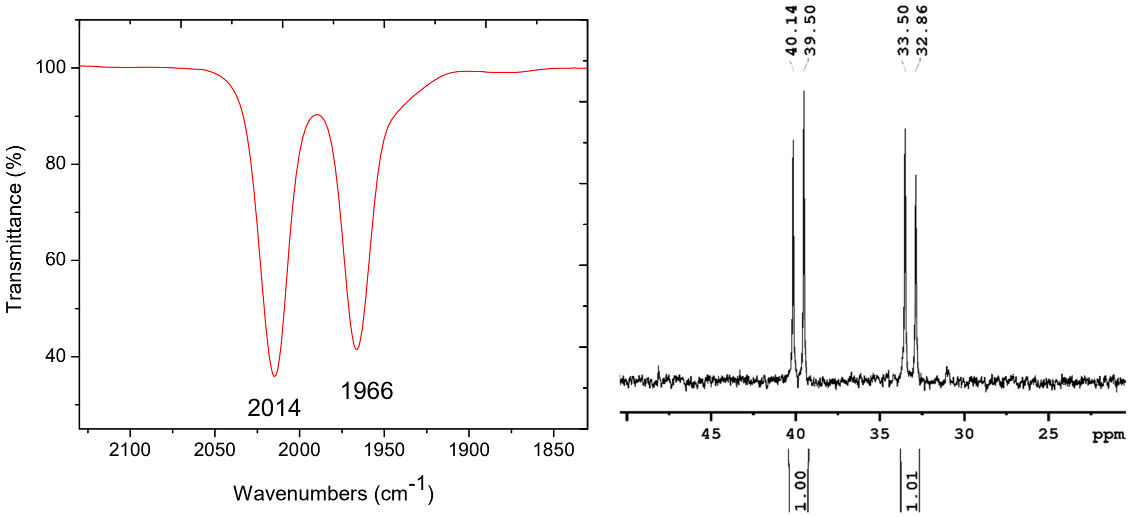

(a) (b)

Figure S1. IR (CH_2Cl_2) (a) and $^{31}P\{^1H\}$ NMR ($CDCl_3$) (b) spectrum of [Fe(κ^2-pdt)$(CO)_2$(κ^2-$P^{Ph}{}_2N^{Ph}{}_2$)] at 25°C.

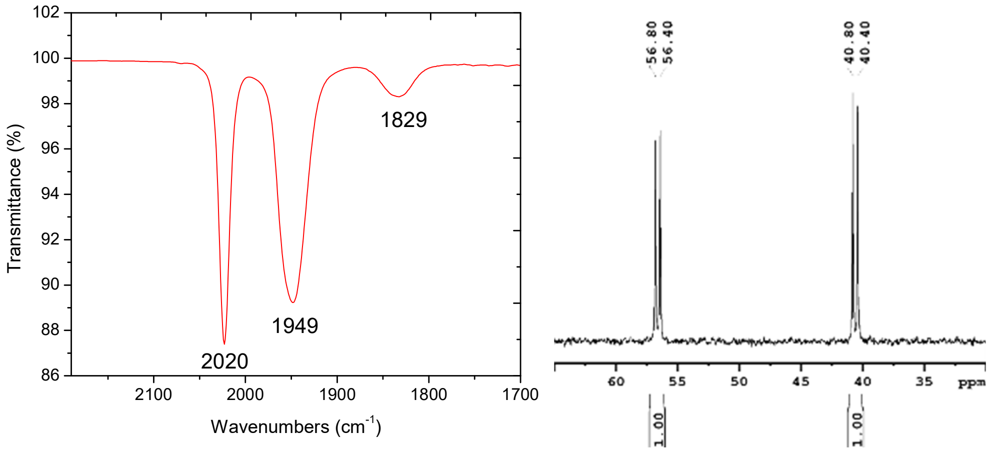

(a) (b)

Figure S2. IR (CH_2Cl_2) (a) and $^{31}P\{^1H\}$ NMR ($CDCl_3$) (b) spectra of **1** at 25°C.

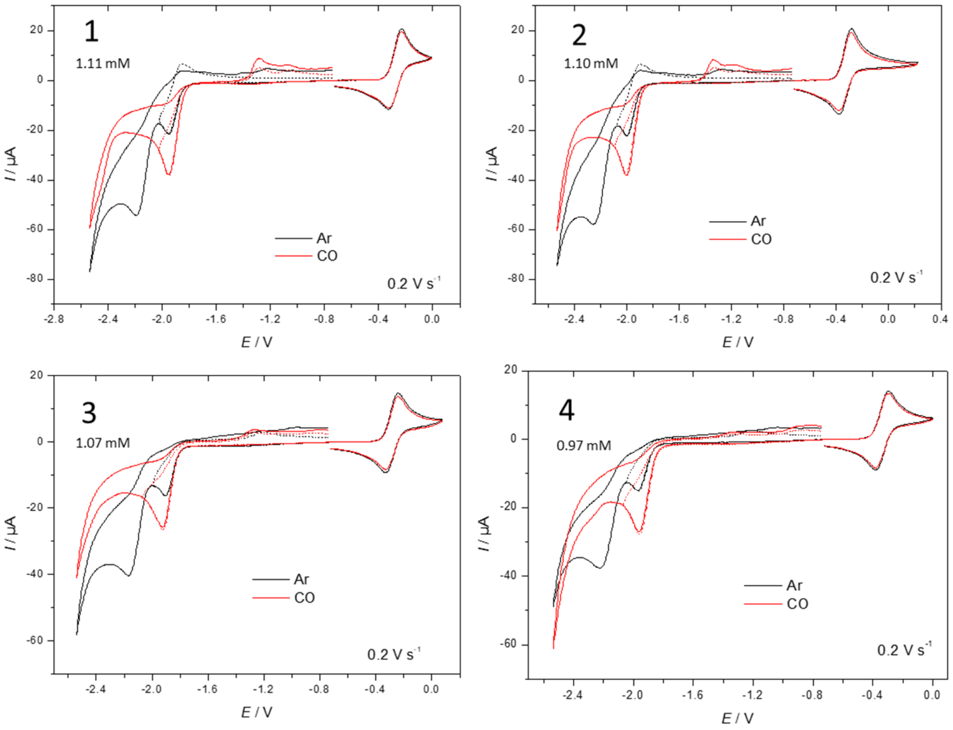

Figure S3. Comparison of CV of **1**-**4** under Ar and CO in CH_2Cl_2-[NBu_4][PF_6] 0.2 M at v = 0.2 V s^{-1}.

Table S1. Crystallographic data of $[Fe_3(CO)_5(\kappa^2\text{-}P^{Ph}{}_2N^{Bn}{}_2)(\mu\text{-adt}^{Bn})(\mu\text{-pdt})]$ (**4**)

Empirical formula	$C_{47}H_{49}Fe_3N_3O_5P_2S_4$
Formula weight	1093.62
Temperature/K	150(2)
Wavelength/Å	0.71073
Crystal system	Monoclinic
Space group	P 1 21 1
a (Å)	15.9368(15)
b (Å)	9.1948(6)
c (Å)	17.7390(18)
α (deg)	90
β (deg)	115.510(12)
γ (deg)	90
V (Å^3)	2346.0(4)
Z	2
ρ_{calc} (Mg.mm^{-3})	1.548
μ (mm^{-1})	1.211
F(000)	1128
Crystal size (mm)	0.22 x 0.07 x 0.02
Crystal color	Intense red
Crystal shape	Plate // (0 0 1)
Range of ϑ (deg)	3.45 to 26.37
Limiting indices	$-19 \le h \le 19, -9 \le k \le 11, -22 \le l \le 20$
Reflections collected / unique	13114 / 8161
R_{int}	0.0991
Completeness to ϑ = 26.37	99.5
Absorption correction	Analytical
Max. / Min. transmission	0.9762 / 0.7764
Refinement method	Full-matrix least-squares on F^2
Data / Restraints / Parameters	8161 / 1 / 577
R_1 [I > 2σ(I)]	0.0671
wR_2 [I > 2σ(I)]	0.0972
R_1 (all data)	0.1186
wR_2 (all data)	0.1283
Goodness-of-fit on F^2	0.969
$\Delta\rho_{max}$, $\Delta\rho_{min}$/e.Å^{-3}	0.913, -0.717

Table S2. Crystallographic data of [$Fe_3(CO)_5(\kappa^2$- $Ph_2NPh_2)(\mu$-pdt$)_2$] (**1**)

Empirical formula	$C_{39}H_{40}Fe_3N_2O_5P_2S_4$
Formula weight	974.46
Temperature/K	150(2)
Wavelength/Å	0.71073
Crystal system	Orthorhombic
Space group	P n a 21
a (Å)	17.7651(16)
b (Å)	11.4986(10)
c (Å)	39.489(9)
α (deg)	90
β (deg)	90
γ (deg)	90
V ($Å^3$)	8067(2)
Z	8
ρcalc (Mg.mm^{-3})	1.605
μ (mm^{-1})	1.398
F(000)	4000
Crystal size (mm)	0.29 x 0.20 x 0.01
Crystal color	Red
Crystal shape	Plate // (0 0 1)
Range of θ (deg)	3.29 to 26.37
Limiting indices	-22 ≤ h ≤ 12, -14 ≤ k ≤ 13, -49 ≤ l ≤ 49
Reflections collected / unique	21073 / 11106
R_{int}	0.1895
Completeness to θ = 26.37	99.7
Absorption correction	Analytical
Max. / Min. transmission	0.9862 / 0.6873
Refinement method	Full-matrix least-squares on F^2
Data / Restraints / Parameters	11106 / 81 / 426
R_1 [I > 2σ(I)]	0.1599
wR_2 [I > 2σ(I)]	0.3550
R_1 (all data)	0.2705
wR_2 (all data)	0.4610
Goodness-of-fit on F^2	1.058
$\Delta\rho_{max}$, $\Delta\rho_{min}$/e.$Å^{-3}$	1.751, -1.860

Table S3. Selected distances and bond angles for **1**.

	**1**		
	Distance (Å)		*Angles (°)*
Fe1-Fe2	2.531(6)	Fe1-Fe2-Fe3	160.3(3)
Fe2-Fe3	2.556(6)	Fe2-C2-O2	157(3)
Fe1-$CO_{terminal}$	1.69(3)	Fe1-C2-O2	125(2)
Fe3-$CO_{terminal}$	1.72(4)-1.86(6)	Fe2-C2-Fe1	77.2(13)
Fe1-$CO_{bridging}$	2.28(3)	Fe1-Fe2-C2	61.5(11)
Fe2-$CO_{bridging}$	1.71(3)	P1-Fe1-P2	82.1(4)
Fe1-P1	2.196(11)		
Fe1-P2	2.218(10)		

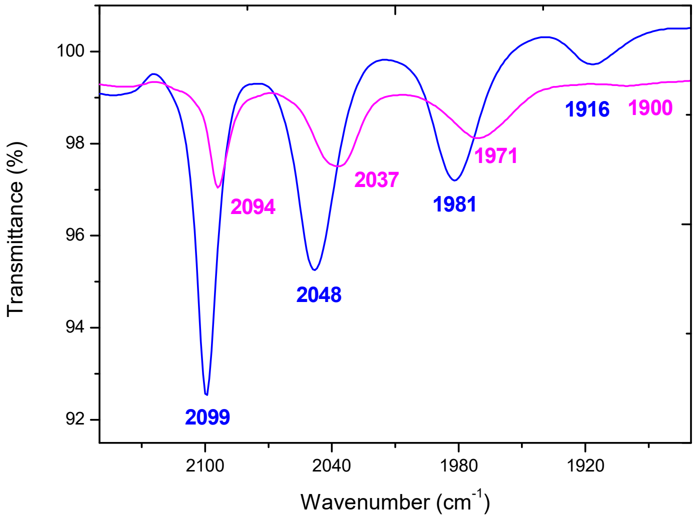

Figure S4. IR spectra of **1** in CH_2Cl_2 in the presence of 6 equiv of CF_3SO_3H (blue curve) and after evolution in the IR cell (purple curve).

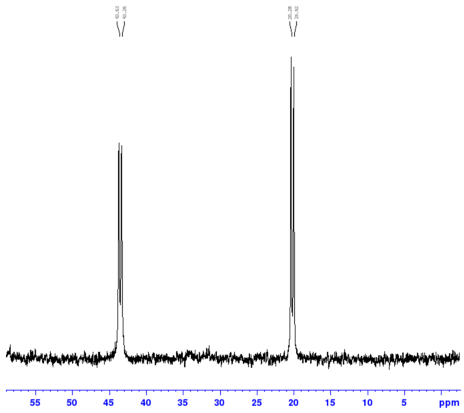

Figure S5. $^{31}P\{^1H\}$ NMR spectrum of **1** at 243 K in the presence of 3 equiv of CF_3SO_3H

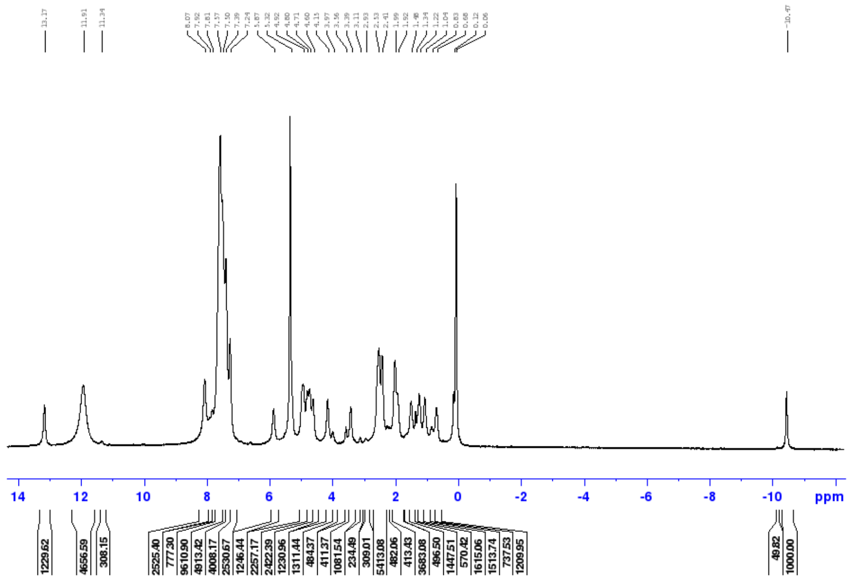

Figure S6. ^{1}H NMR spectrum of **1** at 243 K in the presence of 3 equiv of CF_3SO_3H

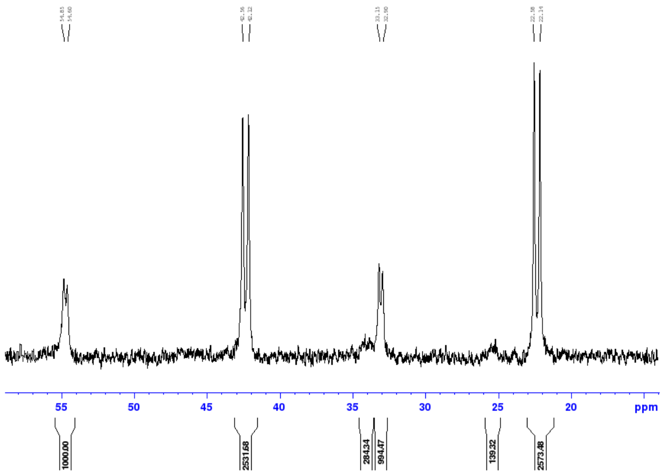

Figure S7. $^{31}P\{^{1}H\}$ NMR spectrum of **2** at 243 K in the presence of 3 equiv of CF_3SO_3H

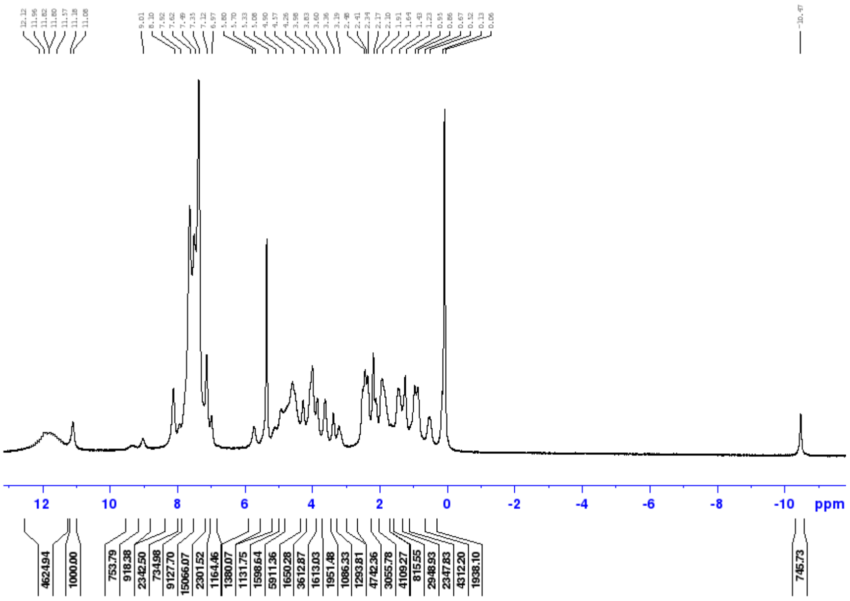

Figure S8. ^{1}H NMR spectrum of **2** at 243 K in the presence of 3 equiv of CF_3SO_3H

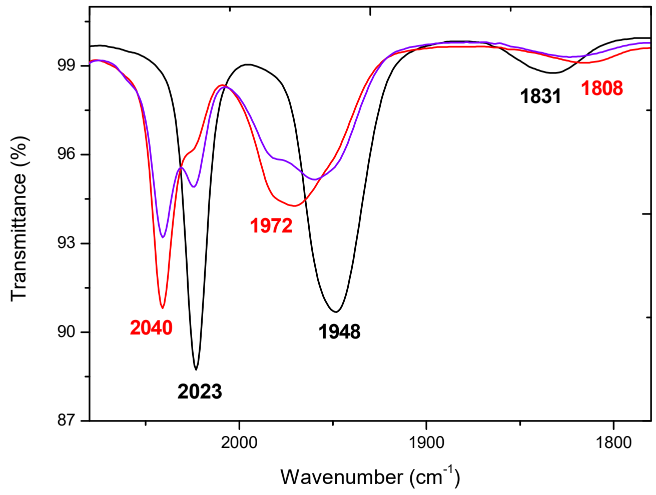

Figure S9. Monitoring in the IR cell of the evolution of **3adt^{Bn}H$^+$** in CH_2Cl_2 after 5 min (red curve) and 10 min (purple curve) ; IR spectrum of **3** in CH_2Cl_2 (black curve).

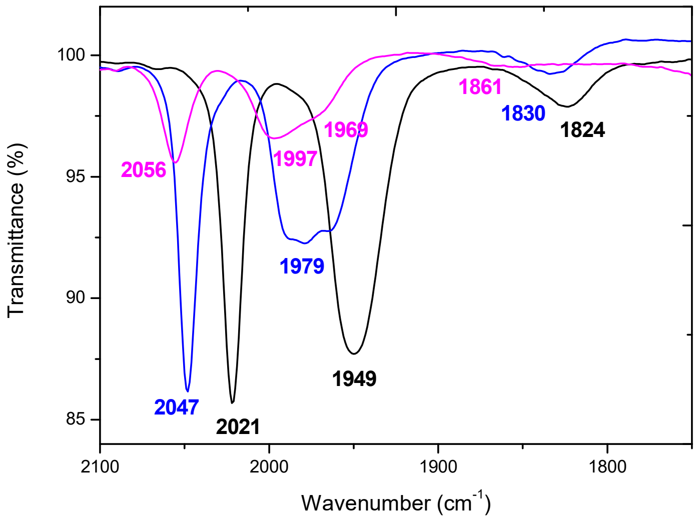

Figure S10. IR (CH_2Cl_2) spectra of complexes **4** in the presence of CF_3SO_3H : 0 equiv (black curve), 2 equiv (blue curve) and 6 equiv (purple curve)
